# *N*-(4-Chloro­pyridin-2-yl)-*N*-(4-methyl­phenyl­sulfon­yl)acetamide

**DOI:** 10.1107/S1600536810048324

**Published:** 2010-11-27

**Authors:** Stefanie Bühler, Dieter Schollmeyer, Wolfgang Albrecht, Stefan Laufer

**Affiliations:** a Eberhard-Karls-University Tübingen, Auf der Morgenstelle 8, 72076 Tübingen, Germany; b University Mainz, Institute of Organic Chemistry, Duesbergweg 10-14, 55099 Mainz, Germany; c c-a-i-r biosciences GmbH, Paul-Ehrlich-Str. 15, 72076 Tübingen, Germany

## Abstract

The crystal structure of the title compound, C_14_H_13_ClN_2_O_3_S, features a three-dimensional network stabilized by inter­molecular C—H⋯O hydrogen bonds between the mol­ecules. The 4-methyl­phenyl­sulfonyl ring forms a dihedral angle of 30.6 (1)° with the 4-chloro­pyridine ring.

## Related literature

For the biological activity of 2-alkyl­amino­pyridinyl or 2-acyl­amino­pyridinyl imidazole derivatives as p38α MAPK inhibitors, see: Laufer *et al.* (2008 ▶, 2010 ▶); Ziegler *et al.* (2009 ▶). For general background to protecting groups, see: Kocieński (2005 ▶). For the preparation of the *N*-protected 4-chloro­pyridine, see: Berliner & Belecki (2005 ▶); Sciotti *et al.* (2005 ▶); Shi & Wang (2002 ▶).
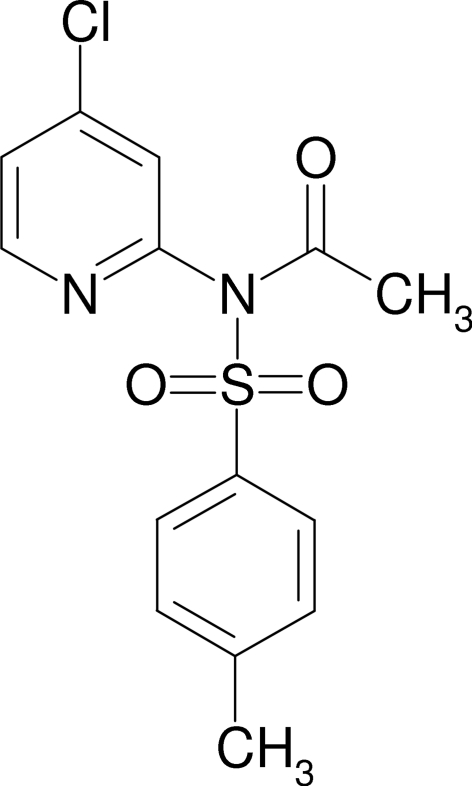


## Experimental

### 

#### Crystal data

C_14_H_13_ClN_2_O_3_S
*M* *_r_* = 324.77
Orthorhombic, 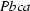

*a* = 12.578 (2) Å
*b* = 7.5460 (8) Å
*c* = 30.194 (3) Å
*V* = 2865.7 (7) Å^3^
*Z* = 8
Cu *K*α radiation
μ = 3.83 mm^−1^
*T* = 193 K
0.35 × 0.35 × 0.25 mm

#### Data collection

Enraf–Nonius CAD-4 diffractometer
Absorption correction: ψ scan (*CORINC*; Dräger & Gattow, 1971 ▶) *T*_min_ = 0.872, *T*_max_ = 0.997
5291 measured reflections
2713 independent reflections
2412 reflections with *I* > 2σ(*I*)
*R*_int_ = 0.079
3 standard reflections every 60 min  intensity decay: 2%

#### Refinement

*R*[*F* ^2^ > 2σ(*F* ^2^)] = 0.049
*wR*(*F* ^2^) = 0.129
*S* = 1.13
2713 reflections
193 parameters
H-atom parameters constrained
Δρ_max_ = 0.44 e Å^−3^
Δρ_min_ = −0.33 e Å^−3^

### 

Data collection: *CAD-4 Software* (Enraf–Nonius, 1989 ▶); cell refinement: *CAD-4 Software*; data reduction: *CORINC* (Dräger & Gattow, 1971 ▶); program(s) used to solve structure: *SIR97* (Altomare *et al.*, 1999 ▶); program(s) used to refine structure: *SHELXL97* (Sheldrick, 2008 ▶); molecular graphics: *PLATON* (Spek, 2009 ▶); software used to prepare material for publication: *PLATON*.

## Supplementary Material

Crystal structure: contains datablocks I, global. DOI: 10.1107/S1600536810048324/bt5410sup1.cif

Structure factors: contains datablocks I. DOI: 10.1107/S1600536810048324/bt5410Isup2.hkl

Additional supplementary materials:  crystallographic information; 3D view; checkCIF report

## Figures and Tables

**Table 1:** Hydrogen-bond geometry (Å, °)

*D*—H⋯*A*	*D*—H	H⋯*A*	*D*⋯*A*	*D*—H⋯*A*
C3—H3⋯O13^i^	0.95	2.46	3.404 (3)	174
C14—H14*B*⋯O10^ii^	0.98	2.50	3.170 (4)	126
C18—H18⋯O9^iii^	0.95	2.46	3.334 (3)	152

Symmetry codes: (i) 
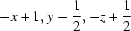
; (ii) 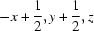; (iii) 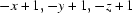.

**Fig. 1.**
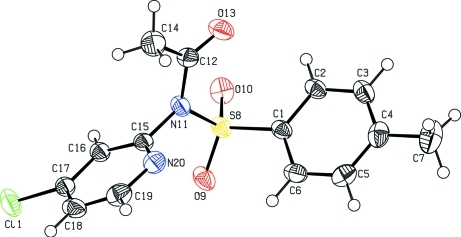
View of compound I. Displacement ellipsoids are drawn at the 50% probability level.

## supplementary crystallographic information

### Comment

In recent years, compounds with the 2-aminopyridine moiety exhibited interesting biological activities like the 2-alkylaminopyridinyl or 2-acylaminopyridinyl imidazole derivatives as p38α mitogen-activated protein kinase (p38α MAPK) inhibitors. The N-protected 4-chloropyridine is an important precursor to block the nucleophilic and basic properties of the amino-group in the C2 position of the pyridine ring. The analysis of the crystal structure shows that the aromatic C18—H-group of the 4-chloropyridine ring of one molecule interacts with the oxygen-atom O9 of the sulfonyl group of another molecule related to the first by centre of symmetry with a distance of H18···O9 2.47 Å. Furthermore, the aromatic C3—H group of the 4-methylphenylsulfonyl ring forms an intermolecular C3—H3···O13_a hydrogen bond (2.46 Å) to the oxygen atom O13 of the acetamide moiety of a third molecule. An additional hydrogen bond was observed between the methyl-group C14—H_3_ of the acetamide moiety and the oxygen-atom O10 of the sulfonyl group of a further molecule, whereas the O10···H14B distance is 2.50 Å. The dihedral angle between the 4-methylphenylsulfonyl ring and the 4-chloropyridine ring is 30.6 (1)°.

### Experimental

Synthesis of chloromethyl methyl ether as a solution of toluene: To a solution of dimethoxymethane (44.3 ml, 0.50 mol, 1 equiv) and Zn(OAc)_2_ (9.2 mg, 0.01%) in toluene (133 ml) was added acetyl chloride (35.5 ml, 0.50 mol, 1 equiv). During the next 15 min, the reaction mixture warmed slowly at T = 318 K, and then cooled to ambient temperature over 3 h. The progress was again monitored until NMR analysis indicated complete conversion. The solution of MOMCl in toluene prepared using this stoichiometry is approximately 2.1 *M*.

Synthesis of *N*-(4-chloropyridin-2-yl)-4-methylbenzenesulfonamide: 2-Amino- 4-chloropyridine (20.1 g, 156 mmol. 1 equiv) and 4-toluenesulfonyl chloride (32.4 g, 168 mmol, 1.1 equiv) were dissolved in dry pyridine (70 ml) and heated at T = 353 K for 5 h. After cooling to room temperature, water was added and the compound *N*-(4-chloropyridin-2-yl)-4-methylbenzenesulfonamide dropped down as a beige solid with high analytical quality, which was filtered off and washed with water (30.6 g, 70.8%).

Synthesis of *N*-(4-chloropyridin-2-yl)-*N*-tosylacetamide: Under a nitrogen atmosphere, *N*-(4-chloropyridin-2-yl)-4-methylbenzene-sulfonamide (20.0 g, 71 mmol, 1 equiv) was added to a suspension of NaH (4.2 g, 104 mmol, 1.5 equiv) in anhydrous THF (200 ml) with stirring. The resulting reaction mixture was stirred for 20 min, and then the solution of methoxymethyl chloride in toluene (52.1 ml, 1.5 equiv) was slowly added. The mixture was stirred for 3 h and then an aqueous saturated solution of NH_4_Cl was added. After separation, the aqueous layer was extracted with EtOAc, dried over Na_2_SO_4 _and evaporated. After treatment with hexane, the compound *N*-(4-chloropyridin-2-yl)- *N*-(methoxymethyl)-4-methylbenzenesulfonamide was obtained as the main product of the reaction (15.8 g, 69.7%) and dropped down as a pale yellow solid, whereas the compound *N*-(4-chloropyridin-2-yl)-*N*-tosylacetamide was isolated from the filtrate as the byproduct (15.4%). Suitable crystals of the byproduct *N*-(4-chloropyridin-2-yl)-*N*-tosylacetamide for X-ray were obtained by slow evaporation at T = 298 K of a solution mixture of EtOAc/hexane.

### Refinement

Hydrogen atoms   were placed at calculated positions with C—H = 0.95 Å (aromatic) or 0.98–0.99 Å (*sp*^3^ C-atom) and refined in the riding-model approximation with isotropic displacement parameters (set at 1.2–1.5 times of the *U*_eq_ of the parent atom).

### Crystal data

**Table:**

C_14_H_13_ClN_2_O_3_S	*F*(000) = 1344
*M**_r_* = 324.77	*D*_x_ = 1.506 Mg m^−^^3^
Orthorhombic, *P**b**c**a*	Cu *K*α radiation, λ = 1.54178 Å
Hall symbol: -P 2ac 2ab	Cell parameters from 25 reflections
*a* = 12.578 (2) Å	θ = 65–69°
*b* = 7.5460 (8) Å	µ = 3.83 mm^−^^1^
*c* = 30.194 (3) Å	*T* = 193 K
*V* = 2865.7 (7) Å^3^	Block, colourless
*Z* = 8	0.35 × 0.35 × 0.25 mm

### Data collection

**Table:**

Enraf–Nonius CAD-4 diffractometer	2412 reflections with *I* > 2σ(*I*)
Radiation source: rotating anode	*R*_int_ = 0.079
graphite	θ_max_ = 70.0°, θ_min_ = 2.9°
ω/2θ scans	*h* = −15→15
Absorption correction: ψ scan (*CORINC*; Dräger & Gattow, 1971)	*k* = 0→9
*T*_min_ = 0.872, *T*_max_ = 0.997	*l* = 0→36
5291 measured reflections	3 standard reflections every 60 min
2713 independent reflections	intensity decay: 2%

### Refinement

**Table:**

Refinement on *F*^2^	Secondary atom site location: difference Fourier map
Least-squares matrix: full	Hydrogen site location: inferred from neighbouring sites
*R*[*F*^2^ > 2σ(*F*^2^)] = 0.049	H-atom parameters constrained
*wR*(*F*^2^) = 0.129	*w* = 1/[σ^2^(*F*_o_^2^) + (0.0592*P*)^2^ + 0.6955*P*] where *P* = (*F*_o_^2^ + 2*F*_c_^2^)/3
*S* = 1.13	(Δ/σ)_max_ = 0.001
2713 reflections	Δρ_max_ = 0.44 e Å^−^^3^
193 parameters	Δρ_min_ = −0.33 e Å^−^^3^
0 restraints	Extinction correction: *SHELXL97* (Sheldrick, 2008), Fc^*^=kFc[1+0.001xFc^2^λ^3^/sin(2θ)]^-1/4^
Primary atom site location: structure-invariant direct methods	Extinction coefficient: 0.00129 (16)

### Special details

**Table:**

Geometry. All e.s.d.'s (except the e.s.d. in the dihedral angle between two l.s. planes) are estimated using the full covariance matrix. The cell e.s.d.'s are taken into account individually in the estimation of e.s.d.'s in distances, angles and torsion angles; correlations between e.s.d.'s in cell parameters are only used when they are defined by crystal symmetry. An approximate (isotropic) treatment of cell e.s.d.'s is used for estimating e.s.d.'s involving l.s. planes.
Refinement. Refinement of *F*^2^ against ALL reflections. The weighted *R*-factor *wR* and goodness of fit *S* are based on *F*^2^, conventional *R*-factors *R* are based on *F*, with *F* set to zero for negative *F*^2^. The threshold expression of *F*^2^ > σ(*F*^2^) is used only for calculating *R*-factors(gt) *etc*. and is not relevant to the choice of reflections for refinement. *R*-factors based on *F*^2^ are statistically about twice as large as those based on *F*, and *R*- factors based on ALL data will be even larger.

### Fractional atomic coordinates and isotropic or equivalent isotropic displacement parameters (Å^2^)

**Table:**

	*x*	*y*	*z*	*U*_iso_*/*U*_eq_	
Cl1	0.26049 (7)	0.59127 (8)	0.519895 (18)	0.0474 (2)	
C1	0.55234 (19)	0.0478 (3)	0.36074 (7)	0.0279 (5)	
C2	0.5457 (2)	−0.0380 (3)	0.31992 (7)	0.0299 (5)	
H2	0.4787	−0.0702	0.3079	0.036*	
C3	0.6385 (2)	−0.0751 (3)	0.29725 (7)	0.0358 (6)	
H3	0.6346	−0.1337	0.2694	0.043*	
C4	0.7371 (2)	−0.0290 (3)	0.31410 (8)	0.0374 (6)	
C5	0.7421 (2)	0.0548 (3)	0.35535 (8)	0.0379 (5)	
H5	0.8093	0.0851	0.3675	0.046*	
C6	0.6503 (2)	0.0940 (3)	0.37862 (7)	0.0342 (5)	
H6	0.6542	0.1519	0.4065	0.041*	
C7	0.8367 (3)	−0.0685 (4)	0.28847 (11)	0.0545 (8)	
H7A	0.8945	−0.0963	0.3091	0.082*	
H7B	0.8562	0.0351	0.2707	0.082*	
H7C	0.8243	−0.1700	0.2689	0.082*	
S8	0.43683 (5)	0.09319 (7)	0.390620 (16)	0.0289 (2)	
O9	0.46215 (17)	0.0989 (2)	0.43669 (5)	0.0394 (4)	
O10	0.35394 (15)	−0.0198 (2)	0.37556 (5)	0.0390 (4)	
N11	0.40466 (17)	0.3056 (2)	0.37834 (6)	0.0295 (4)	
C12	0.38183 (19)	0.3544 (3)	0.33447 (7)	0.0314 (5)	
O13	0.37972 (16)	0.2443 (2)	0.30578 (5)	0.0404 (4)	
C14	0.3638 (3)	0.5483 (3)	0.32551 (8)	0.0451 (6)	
H14A	0.4324	0.6097	0.3245	0.068*	
H14B	0.3201	0.5990	0.3492	0.068*	
H14C	0.3273	0.5623	0.2971	0.068*	
C15	0.41589 (19)	0.4361 (3)	0.41287 (6)	0.0270 (5)	
C16	0.33884 (19)	0.4476 (3)	0.44486 (6)	0.0278 (4)	
H16	0.2787	0.3713	0.4447	0.033*	
C17	0.3522 (2)	0.5750 (3)	0.47748 (7)	0.0305 (5)	
C18	0.4390 (2)	0.6871 (3)	0.47593 (8)	0.0373 (6)	
H18	0.4488	0.7773	0.4975	0.045*	
C19	0.5105 (2)	0.6634 (3)	0.44216 (8)	0.0389 (6)	
H19	0.5704	0.7400	0.4411	0.047*	
N20	0.50149 (17)	0.5390 (3)	0.41048 (6)	0.0343 (4)	

### Atomic displacement parameters (Å^2^)

**Table:**

	*U*^11^	*U*^22^	*U*^33^	*U*^12^	*U*^13^	*U*^23^
Cl1	0.0655 (5)	0.0449 (4)	0.0320 (3)	0.0135 (3)	0.0132 (3)	−0.0045 (2)
C1	0.0379 (13)	0.0247 (10)	0.0211 (9)	0.0010 (9)	−0.0010 (8)	0.0012 (8)
C2	0.0408 (13)	0.0237 (10)	0.0251 (10)	−0.0023 (10)	−0.0025 (9)	−0.0026 (8)
C3	0.0527 (15)	0.0273 (10)	0.0274 (10)	0.0045 (11)	0.0038 (11)	−0.0014 (8)
C4	0.0440 (15)	0.0286 (11)	0.0396 (11)	0.0089 (11)	0.0078 (11)	0.0102 (9)
C5	0.0349 (13)	0.0353 (12)	0.0436 (12)	0.0010 (10)	−0.0063 (11)	0.0064 (10)
C6	0.0455 (15)	0.0296 (11)	0.0275 (10)	0.0013 (10)	−0.0086 (10)	−0.0012 (8)
C7	0.0509 (18)	0.0453 (15)	0.0675 (18)	0.0124 (14)	0.0187 (16)	0.0125 (13)
S8	0.0396 (4)	0.0244 (3)	0.0225 (3)	0.0014 (2)	0.0028 (2)	0.00126 (17)
O9	0.0628 (12)	0.0318 (8)	0.0236 (8)	0.0098 (8)	0.0048 (8)	0.0027 (6)
O10	0.0411 (10)	0.0320 (8)	0.0438 (9)	−0.0061 (8)	0.0062 (7)	0.0011 (7)
N11	0.0382 (11)	0.0257 (9)	0.0244 (8)	0.0029 (8)	0.0009 (7)	−0.0017 (7)
C12	0.0326 (12)	0.0350 (11)	0.0265 (10)	0.0013 (10)	−0.0022 (9)	0.0019 (9)
O13	0.0564 (11)	0.0390 (9)	0.0257 (7)	0.0014 (9)	−0.0055 (7)	−0.0002 (7)
C14	0.0530 (16)	0.0427 (14)	0.0397 (12)	0.0063 (13)	0.0026 (12)	0.0058 (11)
C15	0.0336 (11)	0.0252 (10)	0.0221 (9)	0.0050 (9)	−0.0035 (8)	0.0013 (7)
C16	0.0327 (11)	0.0256 (10)	0.0252 (9)	0.0019 (9)	−0.0014 (8)	0.0014 (8)
C17	0.0397 (13)	0.0281 (10)	0.0235 (9)	0.0091 (10)	−0.0021 (9)	0.0020 (8)
C18	0.0520 (15)	0.0272 (11)	0.0327 (11)	0.0039 (11)	−0.0129 (10)	−0.0039 (9)
C19	0.0379 (13)	0.0321 (12)	0.0467 (13)	−0.0030 (11)	−0.0089 (11)	0.0011 (10)
N20	0.0350 (11)	0.0315 (10)	0.0364 (10)	0.0005 (9)	−0.0008 (8)	0.0025 (8)

### Geometric parameters (Å, °)

**Table:**

Cl1—C17	1.728 (2)	S8—N11	1.6942 (18)
C1—C6	1.390 (3)	N11—C12	1.405 (3)
C1—C2	1.395 (3)	N11—C15	1.441 (3)
C1—S8	1.744 (2)	C12—O13	1.201 (3)
C2—C3	1.382 (4)	C12—C14	1.505 (3)
C2—H2	0.9500	C14—H14A	0.9800
C3—C4	1.385 (4)	C14—H14B	0.9800
C3—H3	0.9500	C14—H14C	0.9800
C4—C5	1.398 (4)	C15—N20	1.329 (3)
C4—C7	1.502 (4)	C15—C16	1.371 (3)
C5—C6	1.384 (4)	C16—C17	1.387 (3)
C5—H5	0.9500	C16—H16	0.9500
C6—H6	0.9500	C17—C18	1.381 (4)
C7—H7A	0.9800	C18—C19	1.371 (4)
C7—H7B	0.9800	C18—H18	0.9500
C7—H7C	0.9800	C19—N20	1.345 (3)
S8—O10	1.4213 (19)	C19—H19	0.9500
S8—O9	1.4276 (16)		
			
C6—C1—C2	120.8 (2)	N11—S8—C1	105.75 (10)
C6—C1—S8	119.24 (16)	C12—N11—C15	121.53 (18)
C2—C1—S8	119.92 (18)	C12—N11—S8	120.24 (15)
C3—C2—C1	118.8 (2)	C15—N11—S8	117.69 (14)
C3—C2—H2	120.6	O13—C12—N11	120.2 (2)
C1—C2—H2	120.6	O13—C12—C14	122.7 (2)
C2—C3—C4	121.5 (2)	N11—C12—C14	117.1 (2)
C2—C3—H3	119.2	C12—C14—H14A	109.5
C4—C3—H3	119.2	C12—C14—H14B	109.5
C3—C4—C5	118.8 (2)	H14A—C14—H14B	109.5
C3—C4—C7	120.5 (2)	C12—C14—H14C	109.5
C5—C4—C7	120.7 (3)	H14A—C14—H14C	109.5
C6—C5—C4	120.8 (2)	H14B—C14—H14C	109.5
C6—C5—H5	119.6	N20—C15—C16	125.0 (2)
C4—C5—H5	119.6	N20—C15—N11	116.06 (19)
C5—C6—C1	119.3 (2)	C16—C15—N11	118.9 (2)
C5—C6—H6	120.4	C15—C16—C17	117.3 (2)
C1—C6—H6	120.4	C15—C16—H16	121.4
C4—C7—H7A	109.5	C17—C16—H16	121.4
C4—C7—H7B	109.5	C18—C17—C16	119.8 (2)
H7A—C7—H7B	109.5	C18—C17—Cl1	120.60 (17)
C4—C7—H7C	109.5	C16—C17—Cl1	119.64 (19)
H7A—C7—H7C	109.5	C19—C18—C17	117.6 (2)
H7B—C7—H7C	109.5	C19—C18—H18	121.2
O10—S8—O9	119.57 (11)	C17—C18—H18	121.2
O10—S8—N11	108.79 (10)	N20—C19—C18	124.4 (2)
O9—S8—N11	103.77 (9)	N20—C19—H19	117.8
O10—S8—C1	109.13 (10)	C18—C19—H19	117.8
O9—S8—C1	108.91 (12)	C15—N20—C19	115.9 (2)
			
C6—C1—C2—C3	−0.5 (3)	O9—S8—N11—C15	3.9 (2)
S8—C1—C2—C3	−178.90 (16)	C1—S8—N11—C15	−110.65 (18)
C1—C2—C3—C4	−0.1 (3)	C15—N11—C12—O13	175.1 (2)
C2—C3—C4—C5	1.0 (3)	S8—N11—C12—O13	3.8 (3)
C2—C3—C4—C7	−179.1 (2)	C15—N11—C12—C14	−3.2 (3)
C3—C4—C5—C6	−1.2 (4)	S8—N11—C12—C14	−174.53 (19)
C7—C4—C5—C6	178.9 (2)	C12—N11—C15—N20	−69.5 (3)
C4—C5—C6—C1	0.6 (3)	S8—N11—C15—N20	102.1 (2)
C2—C1—C6—C5	0.3 (3)	C12—N11—C15—C16	109.6 (2)
S8—C1—C6—C5	178.69 (17)	S8—N11—C15—C16	−78.8 (2)
C6—C1—S8—O10	−158.12 (17)	N20—C15—C16—C17	−0.8 (3)
C2—C1—S8—O10	20.3 (2)	N11—C15—C16—C17	−179.85 (18)
C6—C1—S8—O9	−26.0 (2)	C15—C16—C17—C18	1.9 (3)
C2—C1—S8—O9	152.42 (17)	C15—C16—C17—Cl1	−177.71 (16)
C6—C1—S8—N11	85.00 (19)	C16—C17—C18—C19	−1.6 (3)
C2—C1—S8—N11	−96.60 (19)	Cl1—C17—C18—C19	178.00 (18)
O10—S8—N11—C12	−56.1 (2)	C17—C18—C19—N20	0.2 (4)
O9—S8—N11—C12	175.60 (19)	C16—C15—N20—C19	−0.5 (3)
C1—S8—N11—C12	61.0 (2)	N11—C15—N20—C19	178.49 (19)
O10—S8—N11—C15	132.24 (18)	C18—C19—N20—C15	0.9 (3)

### Hydrogen-bond geometry (Å, °)

**Table:**

*D*—H···*A*	*D*—H	H···*A*	*D*···*A*	*D*—H···*A*
C3—H3···O13^i^	0.95	2.46	3.404 (3)	174
C14—H14B···O10^ii^	0.98	2.50	3.170 (4)	126
C18—H18···O9^iii^	0.95	2.46	3.334 (3)	152

Symmetry codes: (i) −*x*+1, *y*−1/2, −*z*+1/2; (ii) −*x*+1/2, *y*+1/2, *z*; (iii) −*x*+1, −*y*+1, −*z*+1.
